# Polymorphisms of tumor necrosis factor alpha in Middle Eastern population with colorectal cancer

**DOI:** 10.1007/s13277-015-4421-z

**Published:** 2015-11-16

**Authors:** Maha-Abdulla Hamadien, Zahid Khan, Mansoor-Ali Vaali-Mohammed, Ahmad Zubaidi, Khayal Al-Khayal, James McKerrow, Omar Al-Obeed

**Affiliations:** 1 Colorectal Research Chair, Department of Surgery, College of Medicine, King Khalid University Hospital, King Saud University, P.O. Box 7805 (37), Riyadh, 11472 Saudi Arabia; 2 Genomic Research Chair, Department of Biochemistry, College of Science, King Saud University, Riyadh, Saudi Arabia; 3 Skaggs School of Pharmacy and Pharmaceutical Sciences, University of California San Diego, San Diego, La Jolla, CA USA

**Keywords:** Tumor necrosis factor, Polymorphisms, Colorectal cancer

## Abstract

Tumor necrosis factor-alpha (TNF-α) contributes in inflammation and has been implicated in the development of colorectal cancer (CRC). Single nucleotide polymorphisms (SNPs) in *TNF*-*α* promoter could affect the risk of CRC by regulating TNF-α production. This is the first study to investigate *TNF*-*α* SNPs in a Middle Eastern population. In this study, we examined three SNPs in *TNF*-*α* for association with CRC. One hundred CRC patients and 100 controls were genotyped for *TNF*-*α* -308, -238, and -857 using TaqMan allelic discrimination assay. The *TNF*-*α* -238 (G/A) genotype was significantly associated with high risk of CRC (*p* = 0.003552). The distribution of three genotypes of -238 G/A was significantly different between the controls and CRC patients even after Bonferroni’s correction. The AA genotype of -238 G/A SNP was observed at considerably higher proportion (13 %) in CRCs compared to controls (1 %). Additionally, similar to genotypes, the allelic frequencies of -238 G/A were significantly different between the CRC cases and controls (odds ratios (OR) = 7.647, *χ* ^2^ = 18.50, *p* = 0.00002). The genotype frequencies of -308 and -857 were not notably different between the cases and controls. *TNF*-*α* -238A may be useful as a screening marker to identify individuals prior to their acquiring CRC in the Saudi population although, further validations in larger cohorts are needed.

## Introduction

Colorectal cancer (CRC) is one of the most common cancers worldwide. It is the third most common cancer in males and the second in females. Incidence rates of CRC are rapidly increasing in several areas in the world, probably related to a combination of genetic factors as well as lifestyle changes including diet, obesity, and smoking [1–3]. In the Kingdom of Saudi Arabia, it ranks first in incidence among Saudi males and third among Saudi females according to the Saudi Cancer Registry data [4]. Reports from Saudi Arabia suggest that CRC is a more aggressive disease in that population [5, 6].

Inflammation is one of the key factors involved in carcinogenesis, and people with inflammatory bowel disease are at higher risk of CRC [7]. Therefore, polymorphisms of inflammation-related genes have been regarded as potential sources of cancer risk biomarkers [8]. CRC arises from the accumulation of mutations in oncogenes and tumor suppressor genes during tumorigenesis [9]. Biological and epidemiological studies indicated a clear association between chronic inflammation and colorectal cancer [10, 11]. In the recent years, cytokines have received much attention due to their possible role in tumorigenesis [12, 13]. Cytokines are important inflammatory mediators that may act in a regulatory network to directly or indirectly activate downstream signaling pathways key to the development of malignancies [11, 14]. Human predisposition to cancer could be influenced by single nucleotide polymorphisms (SNPs) located in genes encoding cytokines and their receptors, especially in promoter regions [15].

Tumor necrosis factor-alpha (TNF-α) is an important pro-inflammatory cytokine involved in cell growth, differentiation, and apoptosis [16, 17]. It has also been reported to play a critical role in the carcinogenesis [18]. The *TNF*-*α* gene is located on chromosome 6p21.3. Cytokine production is controlled at the transcriptional level, and many promoter polymorphisms have been described [19]. Previous studies indicated that TNF-α-related cellular functions were influenced by polymorphism in the promoter region of the *TNF*-*α* gene [20–22]. Recently, *TNF*-*α* was suggested to be one of the immunomodulatory genes in the progression of sporadic CRC [23].

The aim of our study was to examine the allelic frequencies of three promoter SNPs (-857 C/T, -308 G/A and -238 G/A) in Saudi patients with CRC versus healthy controls, in order to identify any association of these SNPs with susceptibility to CRC. These SNPs were selected as they were the common *TNF*-*α* SNP sites that were examined in other populations besides being included in the SNP500Cancer project that aims to identify and characterize genetic variants in genes important in cancer.

## Material and methods

### Study population

A total of 200 blood samples were obtained from King Khalid University Hospital. These encompassed 100 patients with sporadic colorectal cancer and 100 healthy controls with no history of cancer. Colorectal cancer patients included patients of all ages (median age = 57 years) and all stages of the disease. Patient blood samples were collected preoperatively. None of the patients underwent preoperative irradiation or chemotherapy. Diagnosis of CRC were established by standard diagnostic procedures and confirmed histopathologically. Patients and controls were from Saudi Arabian ethnicity. All controls were gender- and age-matched and recruited from physical examinations after diagnostic exclusion of cancer. The study complied with the requirements, and has been approved by the Ethics Committee of the King Saud University. Patient consent was obtained for this study.

### DNA extraction

Approximately 3 ml of blood samples were collected in vacutainers containing ethylenediaminetetraacetic acid (EDTA) from all subjects enrolled in the study. Genomic DNA was extracted from peripheral blood using the QIAamp DNA Blood Mini Kit (Qiagen, Valencia, CA, USA) and stored at −80 °C until the time of study. After extraction and purification, the DNA was quantitated spectrophotometrically on NanoDrop 8000 (Thermo Scientific, USA), and its purity examined using standard A260/A280 and A260/A230 ratios.

### SNP selection and genotyping

A total of three important *TNF*-*α* SNPs were selected from the SNP500 Cancer project and previous literature [24–26]. SNPs were genotyped using TaqMan allelic discrimination assay as previously described [27]. TaqMan *TNF*-*α* SNP Genotyping Assays having catalogue number 4351379 and assay numbers C_11918223_10 (rs1799724), C_7514879_10 (rs1800629), and C_2215707_10 (rs361525) were acquired from Applied Biosystems. These assays were supplied at 40X concentration. For each PCR, a 5-ng DNA sample was used with 12.5 μL of 2X Universal Master Mix and 1X assay mix in a total of 25 μL reaction volume (Applied Biosystems, Foster City, CA, USA). PCR conditions are as follows: pre-read stage 60 °C for 30 s, hold stage 95 °C for 10 min, PCR stage 95 °C for 15 s and 60 °C for 1 min for 40 cycles, and post-read stage 60 °C for 30 s. All genotypes were determined by endpoint reading on ViiA™ 7 Real-Time PCR System (Applied Biosystems, Foster City, CA, USA). For quality control, 5 % of the samples were randomly selected and subjected to repeat analysis as measure for verification of genotyping procedures. The results were reproducible without any discrepancies.

### Statistical analysis

Comparisons of genotype and allelic frequencies in the CRC group and healthy individuals were calculated according to Pearson’s goodness-of-fit chi-square. Deviation from Hardy-Weinberg equilibrium, *χ* ^2^ values, odds ratios (OR), 95 % confidence intervals (CI), and *p* values were calculated using the web-based programs (http://ihg.gsf.de/cgi-bin/hw/hwa1.pl, http://www.socscistatistics.com). A *p* value of <0.05 was considered as significant. Additionally, for multiple comparisons of the three SNPs in the TNF-α gene that were examined, Bonferroni’s correction was applied with an *α* = 0.0167 considered as significant.

## Results

### Genotype and allele association with CRC risk

To determine the risk of predisposition to colorectal cancer with the genetic variants in the *TNF*-*α* gene, three different loci comprising SNPs rs1800629, rs361525, and rs1799724 were examined in Saudi Arabian patients. All the three SNPs are within 2 kb upstream of the 5′ region of the gene and hence likely to be in the promoter. The study population comprised 100 colorectal cancer cases and the same number of age- and gender-matched normal healthy individuals. The clinicopathological characteristics of CRC patients are presented in Table 1. The distribution of genotype and allele frequencies of the analyzed SNPs along with odds ratio and significance are shown in Table 2. The homozygous ancestral allele was used as a reference to determine the risk of acquiring CRCs associated with the other two genotypes. Of the three SNPs in the *TNF*-*α* gene that were analyzed, statistically significant association with CRC risk was observed only for rs361525. The distribution of the three genotypes of rs361525 as GG, GA, and AA was significantly different between the controls and CRC patients (*χ* ^2^ = 11.28, df = 2, *p* = 0.003552). The genotype frequencies for GG, GA, and AA in CRCs were 0.86, 0.01, and 0.13, whereas in healthy controls, it was found to be 0.97, 0.02, and 0.01, respectively (Table 2). Thus, the AA genotype was observed at significantly higher proportion (13 %) in CRC cases compared to the control group (1 %). It was found that the homozygous AA genotype of the *TNF*-*α* SNP rs361525 posed approximately 14-fold higher risk for developing CRC compared to individuals with the GG genotype (OR = 14.663, *χ* ^2^ = 10.94, *p* = 0.00094). It was also noted that similar to genotypes, the distribution of allelic frequencies of rs361525 was significantly different between the CRC cases and controls (OR = 7.647, *χ* ^2^ = 18.50, *p* = 0.00002). The AA genotype as well as A allele of rs361525 was highly significant even after Bonferroni’s correction for multiple testing.

**Table 1:** Clinicopathological characteristics of colorectal cancer patients

Variable	Number of patients	Percent
Median age (57 years)		
≤57	50	50
>57	50	50
Gender		
Male	64	64
Female	36	36
Primary tumor		
Colon	85	85
Rectum	15	15
Tumor staging (Union internationale contre le cancer (UICC) 2010)		
pT1	4	4
pT2	13	13
pT3	73	73
pT4	10	10
Lymph node status (UICC 2010)		
pN0	68	68
pN1	20	20
pN2	12	12
Clinical staging (UICC 2010) and metastasis		
Stage I	11	11
Stage II	58	58
Stage III	17	17
Metastasis	14	14
Histological grading (UICC 2010)		
G1	1	1
G2	97	97
G3	2	2

**Table 2:** Distribution of *TNF*-*α* SNPs genotype and allele frequencies in colorectal cancer cases and control population

SNP ID	Genotype	CRC *n* (frequency)	Controls *n* (frequency)	OR (95 % CI)	*χ* ^2^ value	*p* value*
rs1800629	GG	67 (0.67)	59 (0.59)	Ref		
	GA	23 (0.23)	23 (0.23)	0.881 (0.448–1.731)	0.14	0.71215
	AA	10 (0.10)	18 (0.18)	0.489 (0.209–1.143)	2.79	0.09464
	Allele					
	G	157 (0.785)	141 (0.705)	Ref		
	A	43 (0.215)	59 (0.295)	0.655 (0.416–1.031)	3.37	0.06644
rs361525	GG	86 (0.86)	97 (0.97)	Ref		
	GA	1 (0.01)	2 (0.02)	0.564 (0.050–6.329)	0.22	0.63808
	AA	13 (0.13)	1 (0.01)	14.663 (1.879–114.420)	10.94	**0.00094**
	Allele					
	G	173 (0.865)	196 (0.98)	Ref		
	A	27 (0.135)	4 (0.02)	7.647 (2.624–22.290)	18.50	**0.00002**
rs1799724	CC	85 (0.85)	85 (0.85)	Ref		
	CT	15 (0.15)	15 (0.15)	1.000 (0.460–2.173)	0.00	1.00000
	TT	0 (0.00)	0 (0.00)	na	na	na
	Allele					
	C	185 (0.925)	185 (0.925)	Ref		
	T	15 (0.075)	15 (0.075)	1.000 (0.475–2.105)	0.00	1.00000

*CRC* colorectal cancer, *OR* odds ratio, *95 % CI* 95 % confidence interval

*p < 0.05 was considered significant and are depicted in bold

The distribution of genotype frequencies of rs1800629 was not statistically significant between the CRC cases and controls (*χ* ^2^ = 2.79, df = 2, p = 0.247381). Although the AA genotype of rs1800629 was found in CRC cases at a lower frequency (0.10) compared to controls (0.18) and conferred about twofold decreased risk against CRCs, this association was not statistically significant (OR = 0.489, *χ* ^2^ = 2.79, *p* = 0.09464) (Table 2). In case of rs1799724 polymorphism, the distribution of genotype and allele frequencies between the CRCs and healthy normal subjects were exactly similar (Table 2). Only the CC and CT genotypes were observed while the TT homozygotes were absent in both the cases and control populations.

### Association of *TNF*-*α* SNPs with CRC risk based on age at disease diagnosis and gender

To investigate whether *TNF*-*α* SNPs rs1800629, rs361525, and rs1799724 are associated with the age at CRC diagnosis, patients were stratified based on the median age at the time of disease diagnosis as ≤57 (*n* = 50) and >57 (*n* = 50) years and the genotype and allele frequencies were compared to the age-matched controls. The distribution of genotype and allele frequencies along with the statistical analysis of the three *TNF*-*α* SNPs in CRC cases and normal control population in the two age groups are shown in Table 3. Similar to the findings in the overall study population, individuals with the AA homozygosity compared to the GG homozygosity in rs361525 were at significantly higher risk of developing CRCs. Additionally, examination of the allelic frequencies of rs361525 suggested that the minor allele A confers significantly higher risk of developing CRCs. However, the genotype as well as allele associations were not affected by the age at disease diagnosis as they were observed in both age groups (Table 3). The genotypes of rs1800629 and rs1799724 did not show any predisposition to CRCs in younger as well as older age group patients. Nonetheless, it was noted that the minor allele A of rs1800629 exerts about twofold protective effect in patients >57 years of age (OR = 0.508, *χ* ^2^ = 4.34, *p* = 0.03726).

**Table 3:** Distribution of *TNF*-*α* SNPs genotype and allele frequencies in colorectal cancer cases and control population based on age

SNP ID	Genotype	CRC *n* (frequency)	Controls *n* (frequency)	OR (95 % CI)	*χ* ^2^ value	*p* value*
≤**57**						
rs1800629	GG	31 (0.62)	30 (0.60)	Ref		
	GA	15 (0.30)	14 (0.28)	1.037 (0.428–2.511)	0.01	0.93606
	AA	04 (0.08)	06 (0.12)	0.645 (0.165–2.516)	0.40	0.52586
	Allele					
	G	77 (0.77)	74 (0.74)	Ref		
	A	23 (0.23)	26 (0.26)	0.850 (0.446–1.621)	0.24	0.62185
rs361525	GG	43 (0.86)	47 (0.94)	Ref		
	GA	00 (0.00)	02 (0.04)	0.218 (0.010–4.677)	1.79	0.18043
	AA	07 (0.14)	01 (0.02)	7.651 (0.904–64.753)	4.64	**0.03126**
	Allele					
	G	86 (0.86)	96 (0.96)	Ref		
	A	14 (0.14)	04 (0.04)	3.907 (1.239–12.323)	6.11	**0.01348**
rs1799724	CC	43 (0.86)	43 (0.86)	Ref		
	CT	07 (0.14)	07 (0.14)	1.000 (0.323–3.095)	0.00	1.00000
	TT	00 (0.00)	00 (0.00)	1.000 (0.019–51.542)	na	1.00000
	Allele					
	C	93 (0.93)	93 (0.93)	Ref		
	T	07 (0.07)	07 (0.07)	1.000 (0.337–2.963)	0.00	1.00000
>**57**						
rs1800629	GG	36 (0.72)	29 (0.58)	Ref		
	GA	08 (0.16)	09 (0.18)	0.716 (0.245–2.089)	0.38	0.53994
	AA	06 (0.12)	12 (0.24)	0.403 (0.135–1.204)	2.74	0.09773
	Allele					
	G	80 (0.80)	67 (0.67)	Ref		
	A	20 (0.20)	33 (0.33)	0.508 (0.267–0.966)	4.34	**0.03726**
rs361525	GG	43 (0.86)	50 (1.00)	Ref		
	GA	01 (0.02)	00 (0.00)	3.483 (0.138–87.708)	1.15	0.28385
	AA	06 (0.12)	00 (0.00)	15.092 (0.826–275.623)	6.52	**0.01068**
	Allele					
	G	87 (0.87)	100 (1.00)	Ref		
	A	13 (0.13)	00 (0.00)	31.011 (1.817–529.290)	13.90	**0.00019**
rs1799724	CC	42 (0.84)	42 (0.84)	Ref		
	CT	08 (0.16)	08 (0.16)	1.000 (0.343–2.913)	0.00	1.00000
	TT	00 (0.00)	00 (0.00)	1.000 (0.019–51.569)	na	1.00000
	Allele					
	C	92 (0.92)	92 (0.92)	Ref		
	T	08 (0.08)	08 (0.08)	1.000 (0.360–2.778)	0.00	1.00000

*CRC* colorectal cancer, *OR* odds ratio, *95 % CI* 95 % confidence interval, *na* not analyzable

*p < 0.05 was considered significant and are depicted in bold

The prevalence of genotype and allele frequencies of the three *TNF*-*α* SNPs that were examined in CRC cases and normal control population according to the gender are presented in Table 4. In both the male as well as female populations, individuals with the AA genotype of rs361525 has significantly higher risk of developing CRCs compared with those having GG homozygosity (male—OR = 10.333, *χ* ^2^ = 6.95, *p* = 0.00837; female—OR = 9.831, *χ* ^2^ = 4.12, *p* = 0.04235). Furthermore, based on the allelic model, the minor A allele of rs361525 exhibited significant association with CRCs albeit not showing gender specificity (Table 4). The *TNF*-*α* polymorphisms rs1800629 and rs1799724 did not show any association with the risk of developing CRCs in males as well as in females as was observed in the overall study population.

**Table 4:** Distribution of *TNF*-*α* SNPs genotype and allele frequencies in colorectal cancer cases and control population based on gender

SNP ID	Genotype	CRC *n* (frequency)	Controls *n* (frequency)	OR (95 % CI)	*χ* ^2^ value	*p* value*
Male						
rs1800629	GG	43 (0.67)	36 (0.56)	Ref		
	GA	12 (0.19)	14 (0.22)	0.718 (0.295–1.746)	0.54	0.46359
	AA	09 (0.14)	14 (0.22)	0.538 (0.209–1.388)	1.67	0.19644
	Allele					
	G	98 (0.766)	86 (0.672)	Ref		
	A	30 (0.234)	42 (0.328)	0.627 (0.361–1.087)	2.78	0.09529
rs361525	GG	54 (0.844)	62 (0.968)	Ref		
	GA	01 (0.016)	01 (0.016)	1.148 (0.070–18.800)	0.01	0.92279
	AA	09 (0.140)	01 (0.016)	10.333 (1.268–84.211)	6.95	**0.00837**
	Allele					
	G	109 (0.852)	125 (0.977)	Ref		
	A	19 (0.148)	03 (0.023)	7.263 (2.092–25.210)	12.73	**0.00036**
rs1799724	CC	54 (0.84)	52 (0.81)	Ref		
	CT	10 (0.16)	12 (0.19)	0.802 (0.319–2.017)	0.22	0.63938
	TT	00 (0.00)	00 (0.00)	0.963 (0.019–49.443)	na	1.00000
	Allele					
	C	118 (0.922)	116 (0.906)	Ref		
	T	10 (0.078)	12 (0.094)	0.819 (0.341–1.970)	0.20	0.65560
Female						
rs1800629	GG	24 (0.67)	23 (0.64)	Ref		
	GA	11 (0.30)	09 (0.25)	1.171 (0.410–3.348)	0.09	0.76787
	AA	01 (0.03)	04 (0.11)	0.240 (0.025–2.307)	1.75	0.18626
	Allele					
	G	59 (0.819)	55 (0.764)	Ref		
	A	13 (0.181)	17 (0.236)	0.713 (0.317–1.603)	0.67	0.41177
rs361525	GG	32 (0.89)	35 (0.97)	Ref		
	GA	00 (0.00)	01 (0.03)	0.364 (0.014–9.258)	0.90	0.34220
	AA	04 (0.11)	00 (0.00)	9.831 (0.509–189.758)	4.12	**0.04235**
	Allele					
	G	64 (0.889)	71 (0.986)	Ref		
	A	08 (0.111)	01 (0.014)	8.875 (1.080–72.920)	5.81	**0.03345**
rs1799724	CC	31 (0.86)	33 (0.92)	Ref		
	CT	05 (0.14)	03 (0.08)	1.774 (0.391–8.055)	0.56	0.45325
	TT	00 (0.00)	00 (0.00)	1.063 (0.020–55.233)	na	1.00000
	Allele					
	C	67 (0.93)	69	Ref		
	T	05 (0.07)	03	1.716 (0.395–7.467)	0.53	0.71900

*CRC* colorectal cancer, *OR* odds ratio and *95* % *CI* 95 % confidence interval, *na* not analyzable

*p < 0.05 was considered significant and are depicted in bold

## Discussion

Inflammation plays an important role in the pathogenesis of CRC. TNF-α is a critical component in the inflammatory pathway whose level is known to be upregulated in CRC including in Saudi patients [28, 29]. Hence, in this study, we investigated the link between genetic variants in the *TNF*-*α* gene promoter and susceptibility to CRC in a Saudi population. Of the three SNPs in the *TNF*-*α* gene that were analyzed, a significant association with CRC was observed only for *TNF*-*α* -238. The other two SNPs, -308 and -857, were not significantly associated. It was found that in our population except for the SNP -857 (rs1799724), the other two SNPs, -308 (rs1800629) and -238 (rs361525), did not follow the Hardy-Weinberg equilibrium (Table 5). A high rate of consanguinity in Saudi Arabia could be a probable cause for this lack of Hardy-Weinberg equilibrium [30, 31].

**Table 5:** Test for deviation from Hardy-Weinberg equilibrium

SNP ID	Genotype	CRC n (frequency)	HWE *P* value	Controls n (frequency)	HWE *p* value
rs1800629	GG	67 (0.67)	0.001442	59 (0.59)	7.804e-06
	GA	23 (0.23)		23 (0.23)	
	AA	10 (0.10)		18 (0.18)	
rs361525	GG	86 (0.86)	1.050e-21	97 (0.97)	9.684e-07
	GA	1 (0.01)		2 (0.02)	
	AA	13 (0.13)		1 (0.01)	
rs1799724	CC	85 (0.85)	0.417474	85 (0.85)	0.417474
	CT	15 (0.15)		15 (0.15)	
	TT	0 (0.00)		0 (0.00)	

*CRC* colorectal cancer, *HWE* Hardy-Weinberg equilibrium

A significantly low frequency of the homozygous “A” allele of *TNF*-*α* SNP -238 in the normal control subjects (0.01), compared to the CRC cases (0.13), implicates this genotype as a risk factor in the Saudi population. The odds of acquiring CRC in individuals with AA homozygosity of SNP -238 were 14-fold higher compared to GG homozygotes. The high level of association between the AA genotype of *TNF*-*α*-238 and CRC existed even after Bonferroni’s correction for multiple comparisons, suggesting this association was not by chance. We hypothesize that the -238A allele of the *TNF*-*α* gene might be inducing higher affinity for transcription factor binding, leading to increased transcription in the tumors. Carriers of the GG genotype of *TNF*-*α*-238 were characterized by low production of TNF-α by the peripheral blood mononuclear cells and in the sputum of chronic obstructive pulmonary disease patients [32, 33]. Additionally, Maxwell and colleagues demonstrated that the GA genotype at *TNF*-*α* -238 was associated with a poorer response to infliximab, an anti-TNF agent in the treatment of rheumatoid arthritis [34].

Recently, Yu et al and Ming et al conducted a comprehensive meta-analysis about *TNF*-*α* -238 polymorphism and gastric cancer susceptibility and reported significant associations in Asian populations [35, 36]. However, another meta-analysis showed no association with gastric cancer in a different patient cohort [37, 38]. A number of studies that investigated the association between *TNF*-*α* -238 promoter polymorphism and risk of CRC have yielded contradictory results [39–41]. A recent meta-analysis on *TNF*-*α*-238 polymorphism did not find a significant association with CRC risk [25]. This difference in the predisposition of the disease related to the SNP could be due to ethnic diversity between populations. Larger studies in the Saudi population as well as other cohorts are required to confirm the association of -238 with the risk of CRC observed in our study.

We found no significant association between *TNF*-*α* -308 polymorphism and CRC patients in the overall analysis. Our results are in agreement with other investigations that did not find a correlation between *TNF*-*α* -308 and sporadic colon cancer in a Spanish population [42], a Hungarian population [43], a Korean population [39], and a Croatian population [23]. In addition, *TNF*-*α* -308 polymorphism was not associated with colon cancer in any European population in a meta-analysis conducted by Fan et al, 2011 [44]. Two previous meta-analyses also reported lack of association of *TNF*-*α* -308 polymorphism with CRC risk [45, 46]. However, a meta-analysis by Min et al reported *TNF*-*α* -308 to be moderately associated with an increased risk of CRC in some Western populations [25].

Our investigation of the risk of CRC associated with -308 polymorphism found an inverse association with the AA genotype in patients >57 years of age as well as in the unstratified subjects. An approximately twofold lower risk of CRC for individuals with the AA genotype was noted compared to the GG genotype. However, this association was not statistically significant. Additionally, in the allelic model, we found that the “A” allele of *TNF*-*α* -308 presents a significant protective effect against CRC in individuals >57 years of age. The mechanism behind this protection cannot be explained and needs further investigation as the “A” allele of *TNF*-*α* -308 is associated with higher expression of the cytokine [47]. The three *TNF*-*α* promoter polymorphisms examined in this study were also included in an investigation to find an association of these SNPs with ulcerative colitis-associated CRC by Garrity-Park et al [26]. The authors found a strong association only with *TNF*-*α* -308 polymorphism and ulcerative colitis-associated CRC both at the genotype and allele levels when compared with ulcerative colitis that did not progress to CRC as controls. The probable cause for the discrepancy between the results by Garrity-Park et al and our study could be due to the differences in the patient study population as they examined a homogenous patient group with ulcerative colitis-associated CRCs while our cases mainly included sporadic CRCs without a history of ulcerative colitis. Further, we examined the DNA from blood samples and hence the genotypes represented in our study subjects were germline while the likelihood of somatic mutations in the DNA extracted from formalin-fixed paraffin-embedded biopsies by Garrity-Park and colleagues cannot be ruled out [26].

Increased local expression of pro-inflammatory cytokines such as TNF-α is often detected at sites of inflammation [48]. Studies exploring the genetic association between *TNF*-*α* promoter SNPs and altered cytokines expression have been demonstrated in vivo and in vitro including a link between -308 SNP and increased TNF-α expression [49–52]. Higuchi et al reported that the T allele of *TNF*-*α* -857 C/T and the A allele of *TNF*-*α* -308 G/A resulted in higher transcriptional activity and an increase in TNF-α cytokine production [53]. Thus, it is suggested that the “A” allele of *TNF*-*α* -238 SNP, highly represented in the CRC cases in our population, may lead to higher expression of TNF-α in the tumors.

The genotype frequencies of -857 were the same in our CRC patients compared to control. In our study, the distribution of the three genotypes in the Saudi population (CC/CT/TT—cases 85/15/0, controls 85/15/0) were different compared to a Croatian population (CC/CT/TT—cases 130/64/6, controls 126/67/7) [23]. Moreover, significant correlation between the TNF-*α* mRNA expression level in CRC tumor tissue, and prevalence of *TNF*-*α* -857 CT and TT genotypes were reported and may be involved in the progression of colon cancer [23]. The differences in the distribution of genotype frequencies of *TNF*-*α* -857 could be due to the ethnic diversity between the two populations.

In conclusion, our study assessed CRC predisposition with genetic variants in the *TNF*-*α* gene in the Saudi population. While the *TNF*-*α* -238 SNP was significantly associated with CRC risk, SNPs -308 and -857 did not correlate with susceptibility to CRC in our population. Because the population size in our study was relatively small, these findings need to be confirmed in larger cohorts. The *TNF*-*α* -238 may be a potential marker for CRC screening in the Saudi population.
